# Synthesis and characterization of turmeric extract‐β‐cyclodextrin inclusion complexes: Metabolite profiling and antioxidant activity

**DOI:** 10.1111/1750-3841.17635

**Published:** 2024-12-28

**Authors:** Chagam Koteswara Reddy, Choong Hwan Lee

**Affiliations:** ^1^ Department of Bioscience and Biotechnology Konkuk University Seoul Republic of Korea; ^2^ Department of Life Sciences GITAM (Deemed to be University) Visakhapatnam India; ^3^ Center of Multidisciplinary Unit of Research on Translational Initiatives (MURTI) GITAM (Deemed to be University) Visakhapatnam India

**Keywords:** *Curcuma longa*, inclusion complex, metabolite profile, UHPLC–LTQ–Orbitrap–MS, β‐cyclodextrin

## Abstract

Turmeric (*Curcuma longa* L.) has gained significant attention for its medicinal properties, yet its therapeutic applications are often limited by low aqueous solubility and susceptibility to environmental factors. This study investigates the formulation of a curcumin‐rich turmeric extract–β‐cyclodextrin inclusion complex (TUE–β‐CD) to enhance its bioactivity and stability. Structural characterization and metabolite profiling of the inclusion complex were conducted using field‐emission scanning electron microscopy, thermogravimetric analysis, X‐ray diffraction, Fourier transform infrared spectroscopy, and ultrahigh‐performance liquid chromatography coupled with LTQ–Orbitrap–mass spectrometry (UHPLC–LTQ–Orbitrap–MS). Results revealed that the inclusion complexes exhibit distinct morphological, spectroscopic, crystalline, and thermal properties compared to both curcumin‐rich TUE and β‐CD, confirming successful encapsulation of turmeric metabolites within β‐cyclodextrin cavities. Results of UHPLC–LTQ–Orbitrap–MS confirmed that β‐CD had differential encapsulation efficiencies for bioactive compounds of turmeric. The results suggest that the inclusion complex significantly improves the thermal stability and bioactivity of turmeric extract, thereby enhancing its potential applications as a functional component in both food and non‐food industries.

## 1 INTRODUCTION

Turmeric (*Curcuma longa* L.), a perennial tuberous plant from the ginger family, is widely distributed in tropical and subtropical regions of Asia, Australia, and South America (Pal et al., 2020; Zhang et al., 2017). Over the years, it has been widely used in various foods as a functional ingredient, serving as spice, flavoring agent, preservative, coloring component, and traditional medicine in India, China, and other East Asian countries (Cozmin et al., 2024; Vardhini et al., 2023). Turmeric is rich in numerous bioactive components, primarily curcumin, curcuminoids, cinnamic acids, terpenoids, and other polyphenols (Aarthi et al., 2020; Vardhini et al., 2023). Traditionally, turmeric has been utilized for its medicinal properties in wound healing and as a remedy for inflammation and abdominal cramps (Ahmed & Gilani, 2009). Furthermore, numerous clinical studies have explored the biological activities of turmeric, including antiaging, antimicrobial, antimutagenic, antioxidant, anticarcinogenic, anti‐inflammatory, and hypolipidemic activities (Kunnumakkara et al., 2023).

The major components of turmeric such as curcuminoids and their derivatives, including curcumin, demethoxycurcumin, and bisdemethoxycurcumin, contribute significantly to its physiological and antioxidant properties (Vardhini et al., 2023). Given their rich nutritional and physiological activities, the use of these components in food, cosmetic, and therapeutic applications is highly promising. However, the applications are often limited by various factors such as bitter taste, poor aqueous solubility, sensitivity to oxidation, limited bioavailability, low thermal stability, and adverse pharmacokinetic profiles (Fang & Bhandari, 2010; Reddy et al., 2020). Notably, these issues can be addressed through encapsulation techniques.

Over the years, several studies have shown that encapsulating bioactive compounds can enhance their aqueous solubility, stability against adverse processing conditions, and mask or reduce their undesirable tastes (Fang & Bhandari, 2010; Reddy et al., 2020). In the food industry, encapsulation and inclusion complexes are efficiently used for various applications such as flavor carriers, delivering agents, and maintaining the stability of bioactive components extracted from different plant parts (Mourtzinos et al., 2007; Reddy et al., 2021). This approach supports the formulation of distinct functional and fortified foods. Cyclodextrins are cyclic oligosaccharides composed of α‐d‐glucose units, formed through the enzymatic modification of starch (Li et al., 2015). At the molecular level, typical cyclodextrins feature a hydrophobic interior and a hydrophilic exterior, enabling the encapsulation of various organic compounds (Cai et al., 2018; Reddy et al., 2020). Moreover, due to their suitable cavity size, biocompatibility, and biodegradability, β‐cyclodextrin (β‐CD) can encapsulate different bioactive compounds to form inclusion complexes that solubilize in water, release slowly, and protect guest molecules (Deng et al., 2022). The encapsulation process can also improve the movement, solubility, bioavailability, and stability of guest molecules under adverse processing conditions (Deng et al., 2022; Fang & Bhandari, 2010).

Recently, to enhance the aqueous solubility, bioavailability, and stability of turmeric metabolites, encapsulation and inclusion complexes of curcumin have been formulated using β‐CD. Arya and Raghav (2021) developed an inclusion complex of curcumin through coprecipitation to improve its aqueous solubility and achieve sustained release. Celebioglu and Uyar (2020) demonstrated that the water solubility and antioxidant activity of curcumin can be enhanced by complex formation with β‐CD via nanofibrous webs. Besides, as an alternative to using a single guest molecule, crude extracts from various plant parts and medicinal herbs—including leaves, flowers, seeds, and roots—have been encapsulated with carrier molecules to address their limitations and enhance the functional activities of these compounds (Kong et al., 2019; Mourtzinos et al., 2007; Reddy et al., 2020; Wang et al., 2011).

However, several studies have reported on the formulation, structural characteristics, and bioactivities of curcumin inclusion complexes with β‐CD. Especially, none of these publications have explored the encapsulation of crude turmeric extract (TUE) with β‐CD, nor examined its metabolite profile, structural characteristics, and bioactivities. Therefore, our study aims to formulate an inclusion complex of curcumin‐rich TUE with β‐CD to enhance its structural characteristics, thermal stability, and antioxidant activities. The structural properties of the formulated inclusion complexes were assessed using conventional techniques such as field‐emission scanning electron microscopy (FE‐SEM), X‐ray diffraction (XRD), thermogravimetric analysis (TGA), and Fourier transform infrared spectroscopy (FT‐IR). Additionally, the metabolite profiling of TUE was conducted using ultrahigh‐performance liquid chromatography–linear trap quadrupole–Orbitrap–tandem mass spectrometry (UHPLC–LTQ–Orbitrap–MS/MS).

## 2 MATERIALS AND METHODS

### 2.1 Materials

The dried rhizomes of turmeric samples were acquired from a traditional local market (Seoul, Korea). β‐CD, 2, 2'‐Azino‐bis(3‐ethylbenzothiazoline‐6‐sulfonic acid) diammonium salt (ABTS), 2, 2‐Diphenyl‐1‐picrylhydrazyl (DPPH), and 6‐hydroxy‐2, 5, 7, 8‐tetramethylchroman‐2‐carboxylic acid (Trolox) was obtained from Sigma‐Aldrich Co. All other solvents, chemicals, and reagents were of analytical grade.

### 2.2 Preparation of samples

The rhizomes of selected turmeric samples were cleaned with distilled water to remove foreign materials and then chopped into small pieces, freeze‐dried, and milled into powder with a pestle in a mortar with liquid nitrogen. All dried powdered samples were stored in freezer for further analysis.

### 2.3 Preparation of curcumin‐rich turmeric extract

The curcumin‐rich TUE was prepared, following the method described by Lee et al. (2019). The turmeric powder (100 g) was mixed with ethanol (1.0 L) and continuously stirred for 12 h at 50°C. After incubation, the sample suspension was centrifuged (3000 *g*, 15 min, and 4°C), and the resultant extract was filtered using a 0.22‐µm filter. The ethanolic extract was dried using a speed vacuum concentrator and stored at −20°C for further analysis.

### 2.4 Preparation of TUE–β‐CD inclusion complexes

#### 2.4.1 Freeze‐drying method

The inclusion complex of TUE and β‐CD was formulated using the freeze‐drying method (Reddy et al., 2020). To formulate inclusion complex, initially, 50 mg of TUE was dispersed in anhydrous ethanol (5 mL). Then, the dispersion was slowly added to aqueous β‐CD solution (0.5 g/50 mL) and incubated at 60°C in a stirring water bath for 5 h. After incubation, the mixture was filtered through a 0.45 µm filter. The soluble filtrate was subjected to freeze‐drying at −55°C for 24 h. After freeze‐drying, the sample was placed in an airtight container at 4°C for further use. The prepared inclusion complex by freeze‐drying method was labeled TUE‐FDM. The recovery yield of inclusion complex was calculated according to the following equation:

$$\begin{array}{ccc} & & \mathrm{Recovery}\;\mathrm{yeild}\;\left(\%\right)\\ & & =\frac{\mathrm{Weight}\;\mathrm{of}\;\mathrm{inclusion}\;\mathrm{complex}}{\mathrm{Weight}\;\mathrm{of}\;\mathrm{TUE}+\mathrm{Weight}\mathrm{ofcy}\mathrm{clod}\mathrm{extr}\mathrm{in}\}\;\times 100} \end{array}$$

#### 2.4.2 Kneading method

The inclusion complex of TUE and β‐CD was formulated in a weight molar ratio of 1:2. In kneading method (Yadav et al., 2016), both TUE and β‐CD were kneaded in a mortar for 30 min with equal volume of ethanol and water to obtain slurry‐like consistency. After kneading, the sample paste was dried for 48 h at room temperature. Later, the sample was ground into a fine powder and placed in an airtight container at 4°C for further use. The prepared inclusion complex by kneading method was labeled TUE‐KNM.

### 2.5 Preparation of physical mixture

To make a physical mixture (TUE‐PMX) of TUE and β‐CD, around 50 mg of TUE was mixed with β‐CD (500 mg) in a mortar and was ground at room temperature until the blend was homogeneous and used as control.

### 2.6 Physicochemical characterization

#### 2.6.1 Morphology analysis of inclusion complexes

The morphological features of each sample were studied through Hitachi S‐4800 FE‐SEM. Tested samples were vacuum coated with palladium to enhance conductivity of prepared samples prior to FE‐SEM analysis.

#### 2.6.2 Powder X‐ray diffractometry (PXRD) analysis

The XRD pattern of each sample was obtained on an X‐ray diffractometer (XPERT MPD) outfitted with a Cu‐*K*_α_ radiation source and operated at 40 kV and 40 mA. The data was recorded at a 2*θ* angle range of 3° to 30° (2*θ*) with a step size of 0.02°.

#### 2.6.3 Thermal analysis of inclusion complexes

Using a thermogravimetric analyzer (TGA, TA Q500) and nitrogen atmosphere (flow rate: 20 mL/min), the TGA curves of the samples were obtained. For TGA analysis, approximately 5 mg of each sample was used and heated at a rate of 10°C/min from 30 to 900°C.

#### 2.6.4 Fourier transform infrared (FT‐IR) analysis

FT‐IR spectra of each sample were obtained on an FT‐IR spectrometer (PerkinElmer Co., Ltd.) over a range of 500–4000 cm^−1^ at room temperature. The potassium bromide disk method was used to obtain FT‐IR spectrum of each sample.

### 2.7 UHPLC–LTQ–Orbitrap–MS/MS analysis

#### 2.7.1 Sample extraction for metabolite profiling

For metabolite extraction, each dried sample (10 mg) was solubilized in methanol (20 mL) using a mixer mill for 15 min, followed by ultra‐sonication for 5 min at 4°C. After sonication, the sample was filtered, vacuum dried, and kept at −20°C for further use (Reddy et al., 2020).

#### 2.7.2 Instrumentation

The metabolite profiling of each sample extract was obtained using a UHPLC–LTQ–Orbitrap–MS/MS. Initially, the sample extract (10 mg/mL) was dispersed in methanol and then stored in glass vial. The UHPLC system is equipped with an autosampler, Vanquish binary pump H system (Thermo Fisher Co.) and a column compartment. Phenomenex KINETEX^®^ C18 column (100 × 2.1 mm^2^, 1.7 µm particle size) was used to chromatographic separation and the operating conditions were adapted from Reddy et al. (2020).

#### 2.7.3 Data processing and multivariate statistical analysis

For data processing, using software Xcalibur, the UHPLC–LTQ–Orbitrap–MS/MS raw data were changed into netCDF (.cdf) format. Using MetAlign software (http://www.metalign.nl), the stabilized peak intensities, precise masses (*m/z*), and retention times were obtained, and the data were handled using SIMCA‐P+ for multivariate statistical analysis via unsupervised principal component analysis (PCA) and supervised partial least squares discriminant analysis (PLS‐DA) (Reddy et al., 2020).

### 2.8 Bioactivity assay analysis of inclusion complexes

In this study, ABTS, DPPH, and ferric reducing antioxidant power (FRAP) assays were performed to determine the in vitro antioxidant activities of sample (10 mg/mL methanol) extracted from inclusion complex and physical mixture inclusion complexes, following the procedures described by Reddy et al. (2020). Antioxidant assays were done triplicate, and the results are expressed as the Trolox equivalent antioxidant capacity (TEAC) concentration (mM) per milligram of sample.

#### 2.8.1 ABTS assay

For the ABTS antioxidant assay, 20 µL of the sample extract was mixed with 180 µL of the ABTS solution in 96‐well plate and incubated for 20 min at room temperature in the dark. The absorbance was measured at 750 nm using a spectrophotometer.

#### 2.8.2 DPPH radical scavenging activity

For the DPPH assay, 20 µL of the sample extract was mixed with 180 µL of the DPPH solution (0.2 mM in ethanol) in 96‐well plate and incubated for 20 min at room temperature in the dark. The DPPH free radical absorbance was measured at 515 nm using a spectrophotometer.

#### 2.8.3 FRAP assay

For the FRAP assay, 10 µL of the sample extract was mixed with 300 µL of the FRAP reagent (300 µL) and incubated at room temperature for 6 min. The absorbance was measured at 570 nm using a spectrophotometer.

### 2.9 Statistical analysis

Statistical analysis was carried out using SPSS 22.0 software (IBM). A one‐way ANOVA test, followed by Duncan's test, was done with a significant level of *p *< 0.05.

## 3 RESULTS AND DISCUSSION

### 3.1 Inclusion complex yield

The recovery yield of formulated inclusion complexes obtained through freeze‐drying and kneading methods was calculated by comparing the amount of dried power recovered to the initial quantities added. The recovery of inclusion complexes as precipitates from aqueous reaction mixtures was found to be highest for the freeze‐drying method (∼81%) and slightly lower for the kneading method (∼76%). The formation of complexes between TUE and β‐CD is influenced by various factors, including the complexation method and the physical conditions of the reaction, such as temperature, time, and the ratio of raw materials.

### 3.2 FE‐SEM analysis

**FIGURE 1:**
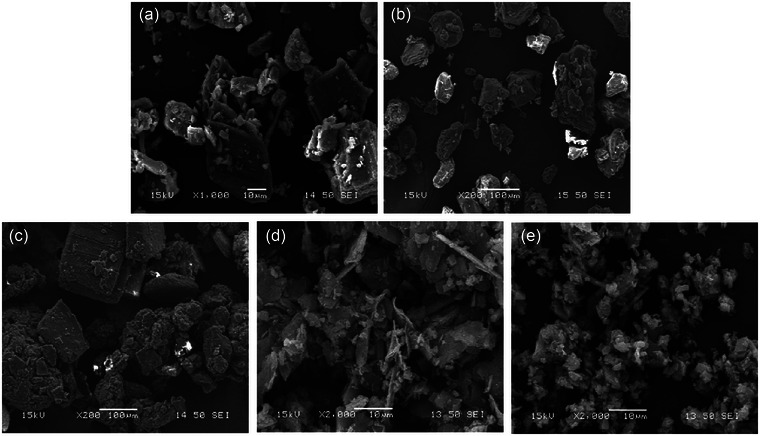
Field‐emission scanning electron microscopy (FE‐SEM) photographs of β‐cyclodextrin (a), ethanolic extract of turmeric (b), physical mixture (c), and inclusion complexes (TUE‐FDM (d) and TUE‐KNM (e)).

FE‐SEM images of curcumin‐rich TUE, β‐CD, and their inclusion complexes are shown Figure 1. The typical structure of β‐CD appeared as irregularly shaped blocky particles varying sizes, and tiny particles visible on the surfaces of the crystals (Figure 1a). In contrast, the pure TUE displayed amorphous, broken, irregular particles as dispersed entities (Figure 1b). The image of the physical mixture (TUE‐PMX) exhibited some similarities with the crystalline structures of the two raw materials, indicating that the metabolites of TUE were adhered to the surface of β‐CD (Figure 1c). This observation suggests that no significant interaction occurred between β‐CD and TUE in this mixture. However, in both types of inclusion complexes, including TUE‐FDM (Figure 1d) and TUE‐KNM (Figure 1e), the unique morphologies of pure TUE and β‐CD were entirely modified, resulting in the formation of tiny, plate‐like structures alongside the crystal particles. It became difficult to distinguish the individual components of the raw materials. These results exhibit a clear interaction between β‐CD and TUE, primarily due to the formation of the inclusion complex. Moreover, the formulated inclusion complexes showed a slightly homogeneous and amorphous appearance. Overall, the FE‐SEM findings strongly indicate the successful formulation of inclusion complexes between β‐CD and TUE.

### 3.3 XRD analysis

**FIGURE 2:**
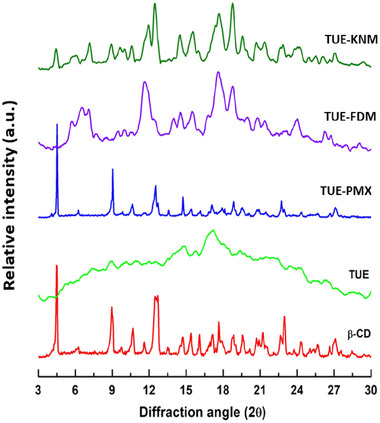
X‐ray diffraction (XRD) patterns of β‐cyclodextrin (β‐CD), ethanolic extract of turmeric (TUE), physical mixture (TUE‐PMX), and inclusion complexes (TUE‐FDM and TUE‐KNM ().

The complex formation of curcumin‐rich TUE with β‐CD is evidenced by the appearance of new diffraction peaks in the XRD patterns, indicating a significant change in the crystalline structure (Gu & Liu, 2020). The XRD patterns of TUE, β‐CD, and their physical mixture (TUE‐PMX) and inclusion complexes (TUE‐FDM and TUE‐KNM) are presented in Figure 2. The results indicate that β‐CD is crystalline, displaying unique diffraction peaks at (*2θ*) values of 4.58°, 9.11°, 10.87°, 12.72°, and 22.96°, which confirms its characteristic cage‐type structure (Reddy et al., 2020). In contrast, TUE exhibits a broad diffraction peak (2*θ*) between 9° and 24°, indicating that TUE primarily existed in an amorphous state. The XRD profile of TUE‐PMX closely resembles that of pure β‐CD, suggesting that the crystalline structure of β‐CD remains unaffected when mixed with TUE. However, the XRD patterns of the inclusion complexes (TUE‐FDM and TUE‐KNM) revealed several new diffraction peaks at 6.54°, 11.68°, 17.58°, 18.69°, 20.79°, and 23.89°. The emergence of these new peaks may indicate a modification of the β‐CD crystalline structure during complex formation, possibly transitioning from a cage‐type to a channel‐type packing arrangement (Okumura et al., 2003). These findings strongly support the formation of inclusion complexes between TUE and β‐CD, as the modification of diffraction patterns reflects significant interactions at the molecular level. This transformation is likely to enhance the stability of the bioactive compounds within the TUE, further improving their potential applications in food and pharmaceuticals.

### 3.4 FT‐IR analysis

**FIGURE 3:**
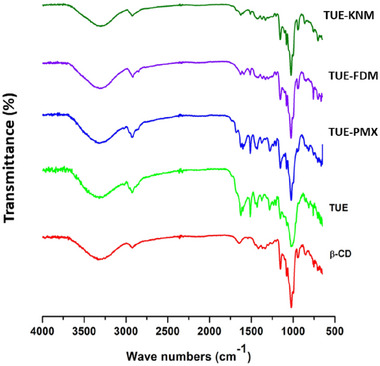
Fourier transform infrared (FT‐IR) spectra of β‐cyclodextrin (β‐CD), ethanolic extract of turmeric (TUE), physical mixture (TUE‐PMX), and inclusion complexes (TUE‐FDM and TUE‐KNM).

The typical FT‐IR spectra of the tested samples are presented in Figure 3. FT‐IR analysis was employed to investigate and confirm the inclusion complex formation between TUE and β‐CD through vibrational changes in FT‐IR spectra, including the reduction, disappearance, or shift of absorption bands, which indicate intermolecular interactions between the raw materials (Escobar‐Avello et al., 2021). The FT‐IR spectrum of β‐CD exhibited typical peaks at 3327 and 2931 cm^−^¹, relating to the stretching vibrations of hydroxyl groups and C–H/CH₂ groups, respectively (Abarca et al., 2016). An absorption band at 1639 cm^−^¹ was associated with the bending vibrations of water molecules trapped in the β‐CD cavities. Moreover, the bands at 1141, 1080, and 1040 cm^−^¹ reflect strong vibrations of C–O, attributed to the ether groups in β‐CD (Reddy et al., 2020). The FT‐IR spectrum of TUE revealed notable bands at 3323, 2925, 1618, 1597, 1436, 1293, 1161, and 1091 cm^−^¹. The band at 3323 cm^−^¹ was linked to hydroxyl group stretching associated with the –OH groups of curcuminoids and their derivatives (Reddy et al., 2020). A prominent band at 1597 cm^−^¹ was attributed to the stretching of the benzene ring in curcumin, whereas the peak at 1340 cm^−^¹ related to C–N vibrations of aromatic amines, and the band at 1293 cm^−^¹ was associated with C = O stretching due to the conjugation of oxygen.

In the FT‐IR spectrum of the TUE‐PMX, the absorption bands were similar to those of β‐CD (Figure 3), confirming that no significant interactions occurred between TUE and β‐CD in this mixture. However, in the spectra of the inclusion complexes (TUE‐FDM and TUE‐KNM), the intensity of the absorption band at 1643 cm^−^¹ changed, and the band at 1597 cm^−^¹ disappeared. This indicates that the components of TUE were successfully embedded within the β‐CD cavity, confirming the formation of an inclusion complex. These results jointly support the conclusion that the interaction between TUE and β‐CD leads to significant changes in their spectral properties, confirming the successful encapsulation of turmeric metabolites within the β‐CD structure.

### 3.5 Thermal properties

**FIGURE 4:**
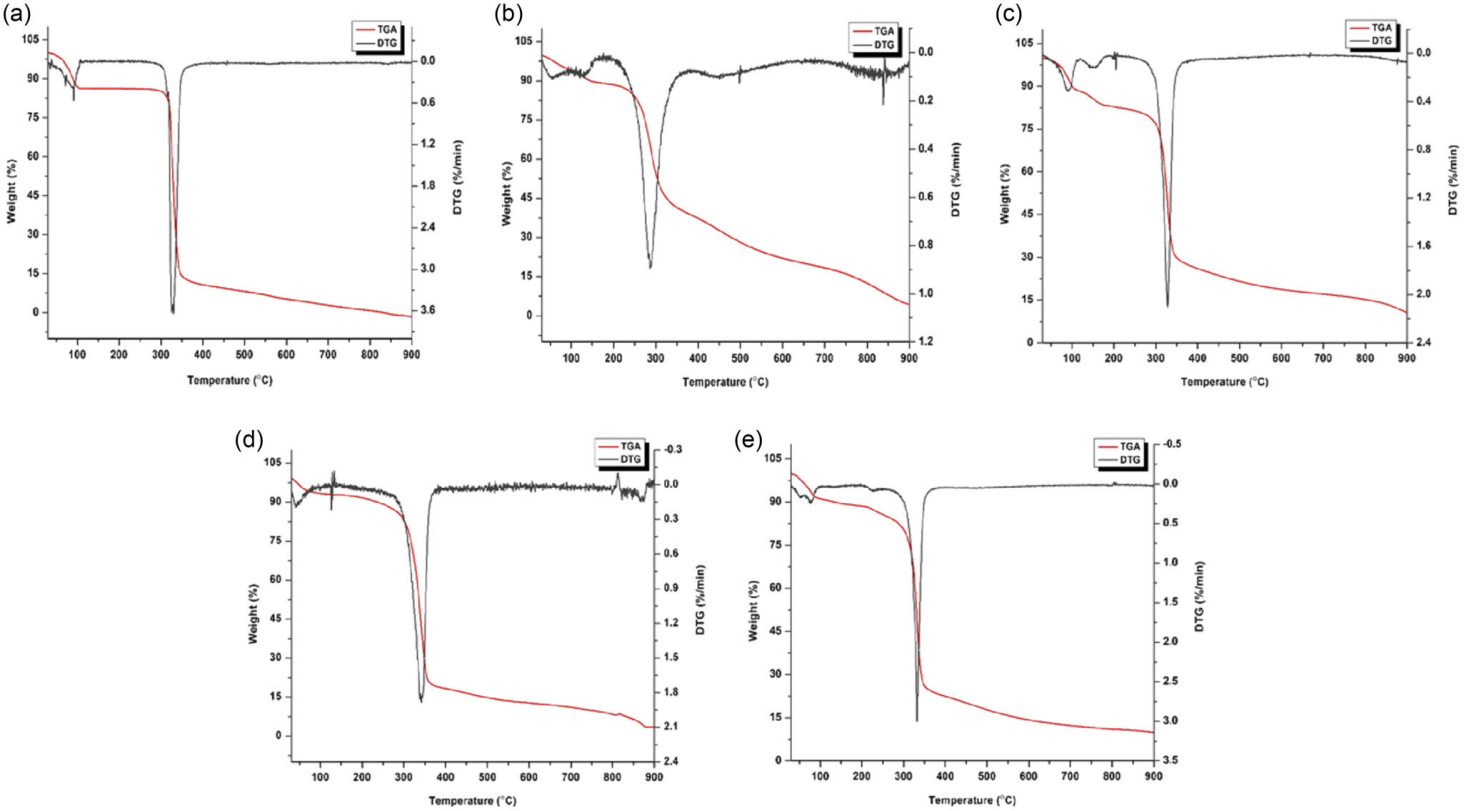
Thermogravimetric analysis (TGA)/DTG thermograms of ethanolic extract of turmeric (a), β‐cyclodextrin (b), physical mixture (c), and inclusion complexes (TUE‐FDM (d) and TUE‐KNM (e)).

The TGA thermograms of TUE, β‐CD, and their inclusion complexes are presented in Figure 4. The thermogram for TUE (Figure 4a) exhibits two prominent peaks corresponding to significant mass loss. The first peak, noticed ∼100°C, indicates a 2.6% loss of mass due to the evaporation of free water molecules, volatile components, and any residual solvent (Sun et al., 2014). The second peak, detected near 130°C, shows an 11%–15% loss of mass, signifying the decomposition of TUE metabolites. Mass loss continues up to 900°C, likely due to the stability of certain structural components within TUE during decomposition. The TGA thermogram of β‐CD (Figure 4b) shows two distinct mass loss events: The first event occurs at approximately 100°C, resulting in a 12.31% weight loss attributed to the evaporation of water molecules located within the β‐CD cavities. The second event occurs at 345°C, corresponding to a 76.83% weight loss due to the decomposition of the macrocyclic structure of β‐CD (Sun et al., 2014).

For the inclusion complexes formulated via freeze‐drying (TUE‐FDM, Figure 4d) and kneading methods (TUE‐KNM, Figure 4e), the TGA thermograms reveal mass loss occurring in three stages. The first stage appears around 100°C, with a 13.05% mass loss due to the evaporation of water from the cavities of the β‐CD. The second stage, indicating the decomposition of the inclusion complexes, is observed at approximately 265°C, with a significant 65.13% loss of mass (Reddy et al., 2021). The final stage, ensuing near 355°C, likely corresponds to the decomposition of carbon, along with organic and inorganic compounds present in the solid particles. To further confirm complex formation, the thermal analysis of the physical mixture of TUE and β‐CD was conducted (Figure 4c). In the TUE‐PMX, the initial mass loss begins around 95°C, consistent with the evaporation of water from β‐CD. The subsequent mass loss phase starts around 345°C, related to the decomposition of β‐CD. Particularly, the decomposition stage for the inclusion complex occurs at nearly 265°C, indicating that the complex formation has altered the thermal stability of β‐CD. These observations confirm that the formation of the inclusion complex affects the thermal stability profile of β‐CD, as evidenced by the lower decomposition temperature compared to the physical mixture. This alteration may enhance the stability of the bioactive compounds in turmeric, making the inclusion complex a promising formulation for food and pharmaceutical applications (Reddy et al., 2020).

### 3.6 Metabolite profiling of inclusion complexes

**FIGURE 5:**
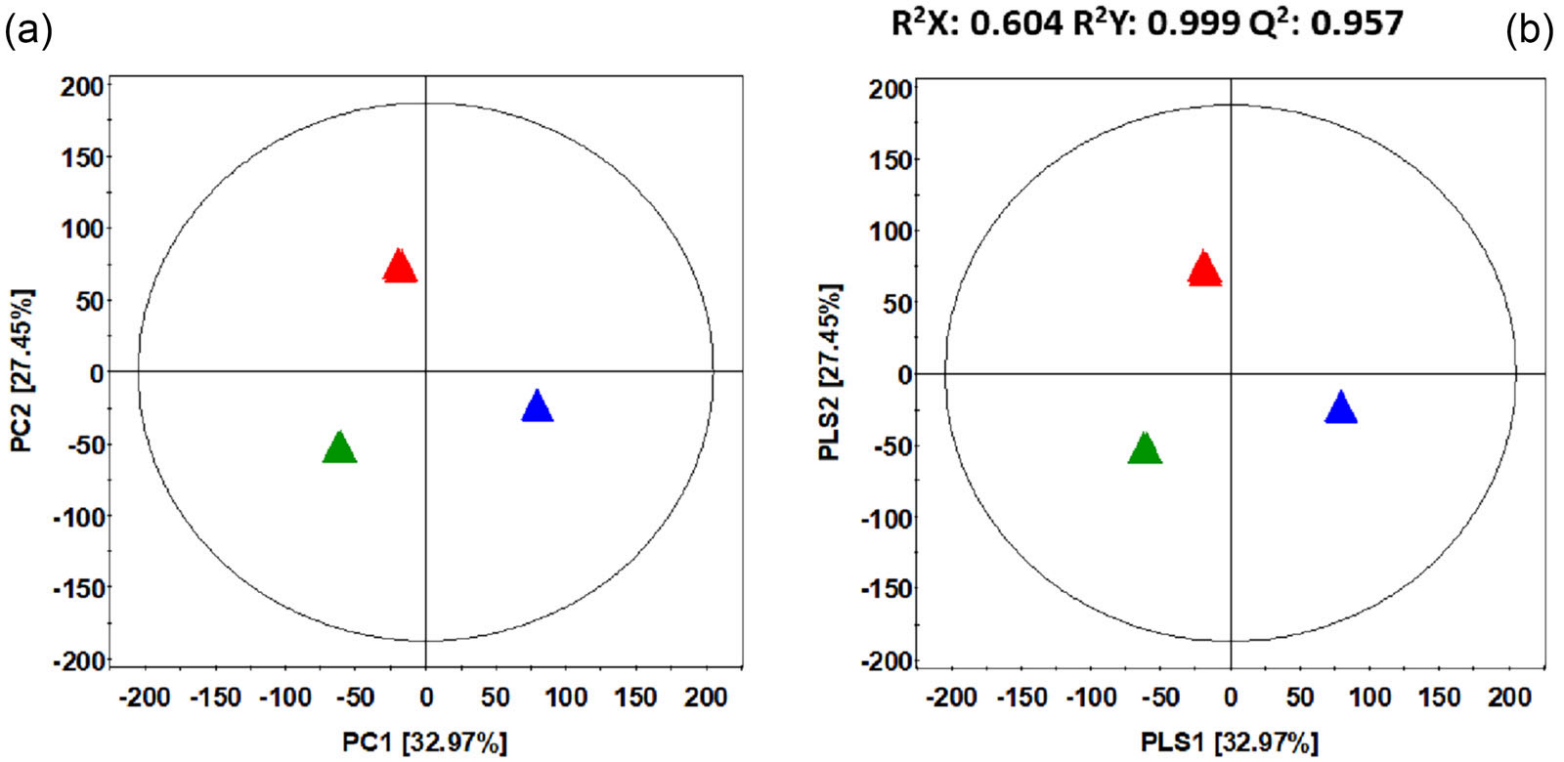
PCA score plot and partial least squares discriminant analysis (PLS‐DA) score plot of physical mixture (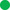
TUE‐PMX), and inclusion complexes (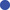
TUE‐FDM, 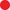
TUE‐KNM). PCA score plot (a) and PLS‐DA score plot (b) derived from UHPLC–LTQ–Orbitrap–MS analysis.

The metabolite profiling was conducted using UHPLC–LTQ–Orbitrap–MS/MS to identify significant variations in metabolites across the different samples. Multivariate analysis through PLS‐DA and PCA models revealed clear distinctions among the samples (Figure 5). The PCA score plot (Figure 5a) showed a distinct pattern for each sample, with PC1 accounting for 32.97% of the variance. Notably, TUE‐FDM was separated from TUE‐KNM along PC2, which explained 27.45% of the variance. A similar trend was observed in the PLS‐DA score plot (Figure 5b).

**FIGURE 6:**
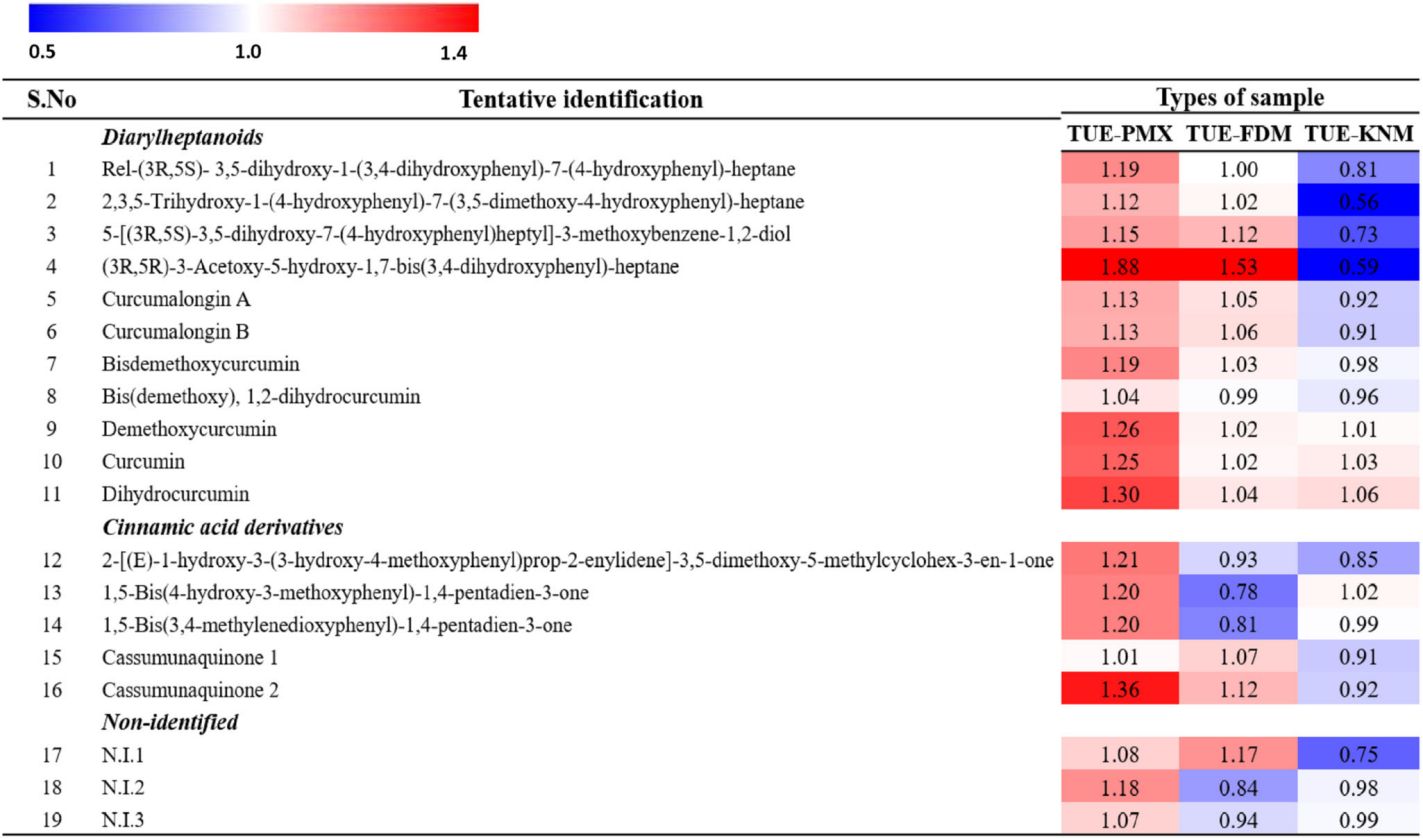
Heat map representation for the relative contents of significantly discriminant metabolites among experimental groups. Metabolites were selected by variable importance in the projection (VIP) value >0.7, p‐value <0.05.

Based on the PLS‐DA results, we identified 19 significantly varied metabolites of TUE, including 11 diarylheptanoids and 5 cinnamic acid derivatives, with VIP values greater than 0.7 and *p*‐values less than 0.05 (Table 1). To further explore the discriminative characteristics of the inclusion complex formed with β‐CD, a heat map was generated to display the differences in the relative volumes of each identified metabolite (Figure 6). The variations in metabolite volumes within the inclusion complexes may be attributed to differences in preparation methods and the ratios of guest (TUE) and host (β‐CD) components (Reddy et al., 2020). Despite the inclusion complexes exhibiting similar types of metabolites post‐complexation (Figure 6), the volumes of these metabolites varied among the samples. Notably, compared to physical mixture, approximately 70%–75% of TUE components were found to be involved in complex formation with β‐CD. These findings suggest that the encapsulation of TUE metabolites within β‐CD not only preserves the diversity of bioactive compounds but also enhances their potential stability and bioavailability. The distinct metabolite profiles observed across different preparation methods underscore the importance of formulation techniques in optimizing the functional properties of TUEs for various applications.

**TABLE 1:** Discriminated metabolites of physical mixture, and turmeric extract‐β‐cyclodextrin (TUE‐β‐CD) inclusion complexes analyzed by UHPLC–LTQ–Orbitrap–MS.

No.	Tentative identification^a^	Ret. (min) ^b^	M.W.^b^	[M − H]^−^	MS fragment pattern (*m/z*)	Molecular formula	mDa (Δppm)	
	***Diarylheptanoids***						
1	Rel‐(3R,5S)‐3,5‐dihydroxy‐1‐(3,4‐dihydroxyphenyl)‐7‐(4‐hydroxyphenyl)‐heptane	5.43	332	331.155	331 > 137 > 119	C_19_H_24_O_5_	3.98
2	2,3,5‐Trihydroxy‐1‐(4‐hydroxyphenyl)‐7‐(3,5‐dimethoxy‐4‐hydroxyphenyl)‐heptane	5.5	392	391.1768	391 > 376 > 151	C_21_H_28_O_7_	2.90
3	5‐[(3R,5S)‐3,5‐dihydroxy‐7‐(4‐hydroxyphenyl)heptyl]‐3‐methoxybenzene‐1,2‐diol	5.52	362	361.1657	361 > 346	C_20_H_26_O_6_	4.62
4	(3R,5R)‐3‐Acetoxy‐5‐hydroxy‐1,7‐bis(3,4‐dihydroxyphenyl)‐heptane	5.73	390	389.1613	389 > 165 > 150	C_21_H_26_O_7_	3.81
5	Curcumalongin A	6.4	352	351.0877	351 > 336 > 308	C_20_H_16_O_6_	2.59
6	Curcumalongin B	6.48	382	381.0976	381 > 366 > 338 > 323	C_21_H_18_O_7_	3.47
7	Bisdemethoxycurcumin	7.65	308	307.0973	307 > 187 > 145	C_19_H_16_O_4_	4.29
8	Bis(demethoxy), 1,2‐dihydrocurcumin	7.56	310	309.1128	309 > 203 > 161	C_19_H_18_O_4_	2.26
9	Demethoxycurcumin	7.79	338	337.1074	337 > 145 > 117	C_20_H_18_O_5_	2.07
10	Curcumin	7.89	368	367.1183	367 > 175 > 160	C_21_H_20_O_6_	3.67
11	Dihydrocurcumin	7.75	370	369.1331	369 > 233 > 175	C_21_H_22_O_6_	3.82
	***Cinnamic acid derivatives***						
12	2‐[(E)‐1‐hydroxy‐3‐(3‐hydroxy‐4‐methoxyphenyl)prop‐2‐enylidene]‐3,5‐dimethoxy‐5‐methylcyclohex‐3‐en‐1‐one	4.94	346	345.1345	345 > 327 > 151	C_19_H_22_O_6_	4.73
13	1,5‐Bis(4‐hydroxy‐3‐methoxyphenyl)‐1,4‐pentadien‐3‐one	5.79	326	325.1087	325 > 307 > 187	C_19_H_18_O_5_	4.31
14	1,5‐Bis(3,4‐methylenedioxyphenyl)‐1,4‐pentadien‐3‐one	6.36	322	321.0773	321 > 293 > 265	C_19_H_14_O_5_	5.33
15	Cassumunaquinone 1	7.18	323	323.0926	323 > 186 > 143	C_19_H_16_O_5_	5.23
16	Cassumunaquinone 2	7.28	353	353.1028	353 > 217 > 173	C_20_H_18_O_6_	2.54
	***Non‐identified***						
17	N.I.1	5.77	434	433.1875	433 > 387 > 181 > 166	C_23_H_30_O_8_	–
18	N.I.2	6.11	312	311.129	311 > 205 > 99	C_19_H_20_O_4_	–
19	N.I.3	7.42	756	755.2678	755 > 579 > 443	C_49_H_40_O_8_	–

^a^ Tentative metabolites based on VIP > 0.7 and *p *< 0.05.

^b^ RT and MW indicates retention time and molecular weight, respectively.

### 3.7 Antioxidant activities of inclusion complexes

**FIGURE 7:**
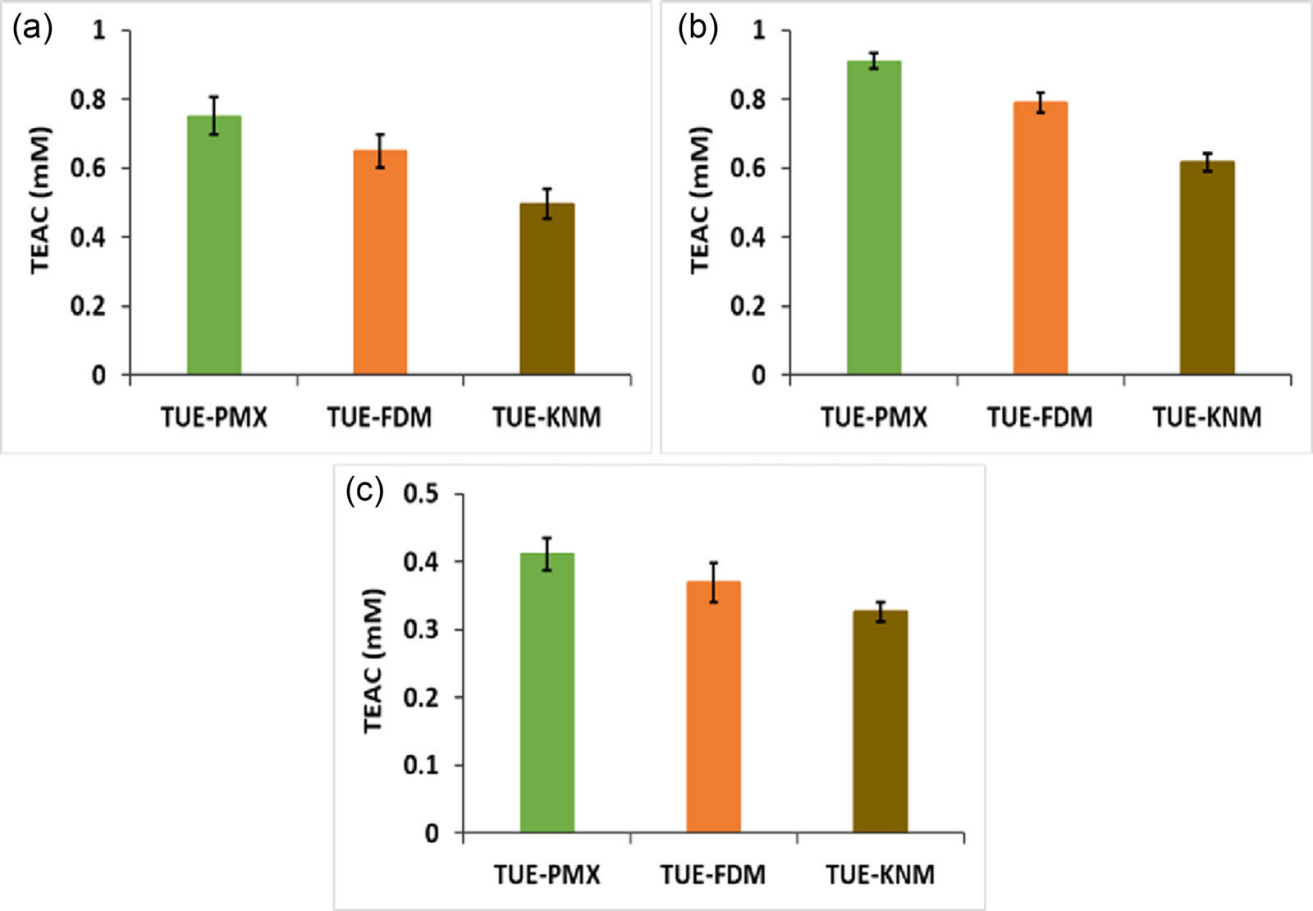
Bioactivity assays of physical mixture and inclusion complexes: (a) DPPH, (b) ABTS, and (c) FRAP.

The antioxidant activities of the inclusion complexes were evaluated using ABTS, DPPH, and FRAP assays, with results presented in Figure 7a–c. Pure TUE exhibited high antioxidant values in all assays, with total antioxidant capacity values of 0.95, 0.98, and 0.96 mM TEAC for the ABTS, DPPH, and FRAP assays, respectively. These high antioxidant activities can be attributed to the substantial volume of bioactive metabolites present in the pure extract. In contrast, β‐CD showed no significant antioxidant activity, reinforcing its role primarily as a carrier rather than an active agent in these formulations. The antioxidant activity of the formulated inclusion complexes was ranked as follows: TUE‐KNM < TUE‐FDM < TUE‐PMX. This hierarchy indicates that the antioxidant activity of the inclusion complexes is significantly influenced by the volume of TUE metabolites, including curcumin, bisdemethoxycurcumin, demethoxycurcumin, and dihydrocurcumin, Moreover, the differences in bioactivity among the inclusion complexes can be attributed to both the quantity of guest molecules and the specific preparation method used for complex formation. These findings underscore the importance of formulation techniques in enhancing the bioactive potential of TUEs, suggesting that optimizing the encapsulation process can lead to improved antioxidant properties.

## 4 CONCLUSIONS

In this study, we prepared the inclusion complex of curcumin‐rich TUE with β‐CD using freeze‐drying and kneading methods. Various conventional techniques, including FE‐SEM, XRD, TGA/DTG, and FT‐IR, confirmed the complex formulation, supporting that the metabolites of TUE were effectively encapsulated within the cavities of β‐CD. The UHPLC–LTQ–Orbitrap–MS/MS findings further indicated that β‐CD exhibits selective inclusion capacity for TUE metabolites, including curcumin, bisdemethoxycurcumin, demethoxycurcumin, and dihydrocurcumin, highlighting the specific interactions between the raw materials. In addition, the stability and antioxidant activity of TUE were significantly enhanced following the complex formulation by freeze‐drying method, compared to kneading method. These results underscore the potential of TUE–β‐CD inclusion complexes to improve the applications of TUEs in both food and nonfood industries, offering a promising approach to enhance the bioavailability and functionality of bioactive compounds.

## AUTHOR CONTRIBUTIONS

**Chagam Koteswara Reddy**: Conceptualization; investigation; data curation; validation; visualization; software; writing—review and editing. **Choong Hwan Lee**: Project administration; supervision; resources; writing—review and editing.

## CONFLICT OF INTEREST STATEMENT

The authors declare that they have no conflicts of interest.
